# CSFPR-RTDETR-CR: A Causal Intervention Enhanced Framework for Infrared UAV Small Target Detection with Feature Debiasing

**DOI:** 10.3390/s26061941

**Published:** 2026-03-19

**Authors:** Honglong Wang, Lihui Sun

**Affiliations:** School of Management Sciences and Information Engineering, Hebei University of Economics and Business, Shijiazhuang 050061, China; whl_19990408@163.com

**Keywords:** infrared UAV, small target detection, feature debiasing, causal reasoning, causal data augmentation, counterfactual reasoning, causal attention mechanism

## Abstract

Infrared UAV small target detection is critical in areas such as military reconnaissance, disaster monitoring, and border patrol. However, it faces challenges due to the small size of targets, weak texture, and complex backgrounds in infrared images. Existing deep learning-based object detection models often learn spurious correlations between targets and their backgrounds. This leads to poor generalization and higher rates of false positives and missed detections in complex scenes. To overcome feature bias and improve performance, this paper proposes an enhanced detection framework based on causal reasoning. The framework builds on the advanced CSFPR-RTDETR detector. Guided by the principles of structural causal models, it explicitly separates causal and non-causal features in the feature space. Feature debiasing is achieved through a three-path approach. First, a causal data augmentation module is introduced. It applies frequency perturbations drawn from a Gaussian distribution to non-causal features. This strengthens the model’s robustness against mixed disturbances. Second, a counterfactual reasoning module is integrated into the backbone network. This module generates counterfactual samples to intervene in the feature distribution, helping the model identify and utilize causal features more effectively. Third, a causal attention mechanism module is added to the encoder. By distinguishing and weighting causal and non-causal features, it guides the model to focus on features that are essential for detecting targets. Experiments on the HIT-UAV public dataset show that the proposed framework improves mAP@50 by 5.6% and mAP@50:95 by 1.8%. Visualization analysis further confirms that the framework enhances feature discrimination and overall detection performance.

## 1. Introduction

Infrared UAV systems, valued for their all-weather and highly discreet observation capabilities, have become essential tools for modern low-altitude monitoring. They play a critical role in military reconnaissance, disaster response, border patrol, and other applications. Detecting small targets in infrared images captured by UAVs is vital for achieving real-time awareness and accurately localizing weak or tiny targets on the ground or at low altitudes. The success of this detection directly affects the accuracy of mission decisions and the efficiency of operational responses. However, this task is highly challenging. Infrared images often have low contrast, limited texture information, and substantial noise. Additionally, UAVs capture images from varied perspectives, backgrounds are complex, and target sizes are extremely small. These factors make it easy for target features to be obscured within the scene [1].

Furthermore, although deep learning-based object detection models have achieved significant progress in general scenarios, they face specific limitations in detecting small objects in infrared UAV imagery. These models [2,3] are fundamentally data-driven and often learn spurious statistical associations between targets and the contexts present in the training data, rather than discovering the stable causal mechanisms behind these associations [4]. As a result, the models have limited generalization ability when backgrounds change, making them prone to scene-dependent bias. For instance, a model may wrongly associate certain terrain textures or lighting conditions with a target category, leading to substantial performance degradation in real-world deployments with varying backgrounds.

In recent years, causal reasoning theory [5,6,7,8,9] has been applied to the field of computer vision [10]. The core idea is to distinguish between causal and non-causal features in data to reduce bias, providing a theoretical foundation for feature debiasing [11]. In general object detection, researchers have explored the use of structural causal models for backdoor adjustment to enhance robustness [12] or have designed causal attention mechanisms to reduce background interference [13]. Similarly, in remote sensing image interpretation, causal reasoning has been investigated to improve the robustness of ship [14] and pedestrian [15] detection. In weakly supervised target localization, multi-dimensional causal intervention has been shown to effectively mitigate co-occurrence bias between categories [16]. Although these studies demonstrate the potential of causal reasoning, several limitations remain for its application in detecting small targets in infrared UAV imagery. First, current research primarily focuses on visible-light images and lacks comprehensive causal modeling tailored to infrared data. Second, there is no established, unified end-to-end framework specifically designed to mitigate feature bias and improve detection performance.

To address the aforementioned challenges, this paper introduces a comprehensive learning paradigm called the Triple Path Causal Intervention Framework. This framework enhances the performance of small target detection in infrared UAV imagery by mitigating feature biases. The main contributions of this work are as follows:

(1) Theoretical Modeling: A structural causal model is developed for infrared UAV small target detection, in which causal and non-causal features are formally defined. This establishes a theoretical foundation for feature debiasing within the model.
(2) Framework Design: A novel framework for debiased detection based on triple-path causal intervention is proposed. It uses the advanced CSFPR-RTDETR object detector as the baseline and integrates three components: causal data augmentation, counterfactual reasoning, and a causal attention mechanism. This integration enables end-to-end feature debiasing from input to output.
(3) Experimental Verification: Experiments on the HIT-UAV public dataset demonstrate that the proposed framework significantly improves both mAP@50 and mAP@50:95. Visualization analyses further confirm the framework’s effectiveness in enhancing feature discrimination, reducing feature bias, and improving overall detection performance.

The structure of the remainder of this article is as follows. Section 2 provides a detailed description of the proposed Triple Path Causal Intervention Framework. Section 3 presents the experimental setup and implementation details. Section 4 systematically reports and analyzes the experimental results. Finally, Section 5 concludes the paper and discusses future research directions.

## 2. A Triple Path Causal Intervention Framework

### 2.1. CSFPR-RTDETR-CR Model

**Figure 1:**
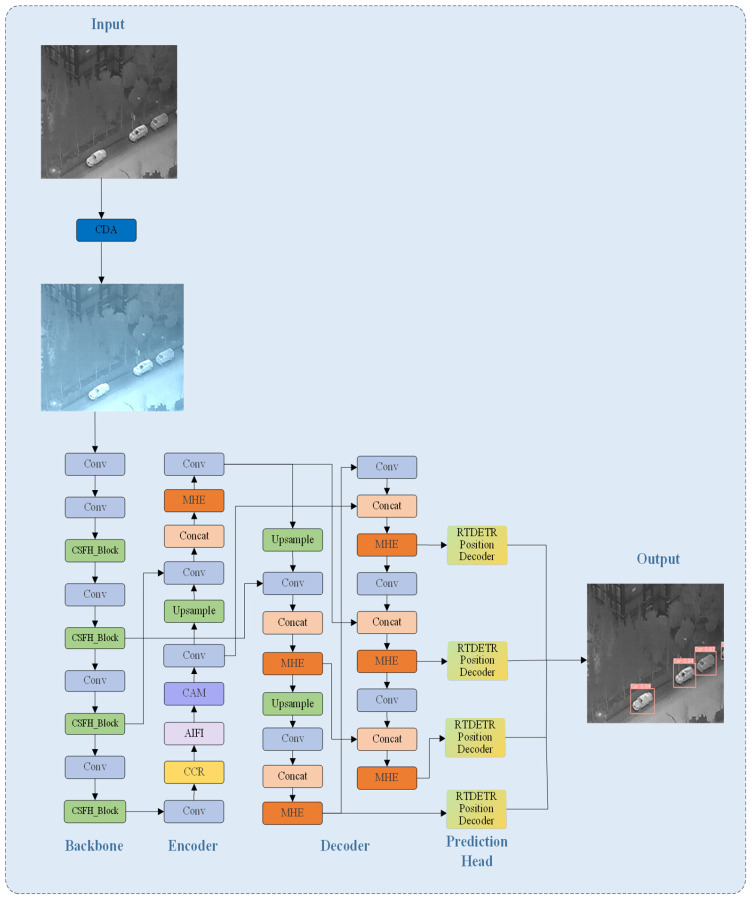
Structure diagram of the CSFPR-RTDETR-CR model.

As illustrated in Figure 1, this paper introduces the CSFPR-RTDETR-CR model, which serves as a specific implementation of a Causal Intervention Enhanced Framework for Infrared UAV Small Target Detection with Feature Debiasing. The model’s overall architecture consists of six key components: the Input stage, the Backbone network, the Encoder, the Decoder, the Prediction Head, and the Output stage. Together, these components form a comprehensive causal intervention chain that spans from data preprocessing to the generation of detection results. At the Input stage, a Causal Data Augmentation (CDA) module applies Gaussian distribution-based frequency-domain perturbations to the original infrared images. This process increases the variation of non-causal features and enhances the model’s robustness against background disturbances. In the Backbone, a Counterfactual Reasoning (CCR) module is integrated. By constructing counterfactual samples to intervene in the feature distribution, the model is guided to identify and reinforce causal features relevant to the targets. Additionally, a Causal Attention Mechanism (CAM) module is incorporated into the Encoder. This module explicitly distinguishes and weights causal and non-causal features, encouraging the model to focus on the causal information essential for target detection. The advanced semantic features then undergo multi-scale fusion and spatial structure reconstruction within the Decoder. Finally, the Prediction Head generates target bounding boxes along with confidence scores and category information. This architecture embeds the triple-path intervention mechanism comprising causal data augmentation, counterfactual reasoning, and the causal attention mechanism throughout the feature processing pipeline. The design aims to enhance the model’s generalization performance in complex infrared UAV scenarios.

### 2.2. Structural Causal Model

**Figure 2:**
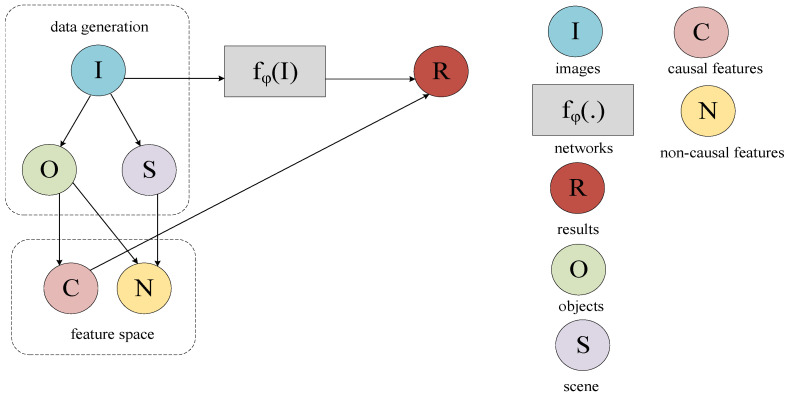
SCM structure diagram.

As illustrated in Figure 2, this paper constructs a Structural Causal Model (SCM) [17] to clarify the causal relationships among key variables in infrared UAV small target detection. The model captures the intrinsic causal structure among images, features, and detection results using a directed acyclic graph. Within this framework, each variable has a specific physical meaning: I denotes infrared images; O represents small targets; C refers to causal features (such as shape) that are influenced by O and serve as critical evidence; N indicates non-causal features that have no causal connection with O (such as background S); fφ(·) denotes deep learning-based object detection models; and R represents the output results of the detectors. Furthermore, following the principle of causal invariance [18], the formal definitions of C and N in this paper are consistent with Pearl’s classical causal reasoning framework. Accordingly, this work primarily focuses on uncovering the influence of causal features on detection results and elucidating the mechanisms by which non-causal features introduce deviations. This provides a theoretical foundation for feature debiasing in subsequent models.

The theoretical framework of the SCM presented in this paper is structured as follows: (1) O ← I → S: The image I is jointly generated by the target O and the background S. (2) O → N ← S: The non-causal feature N is influenced by both O and S, representing a major source of spurious statistical associations. The CDA, CCR, and CAM modules introduced in this paper are specifically designed to block this pathway. (3) O → C → R: The fundamental characteristics of O (such as shape) influence the causal feature C, which in turn affects the prediction R. (4) C ← O → N: As a common cause, O simultaneously generates both C and N, which together constitute the feature space of the model. (5) I → fφ(I) → R: Through the mapping of fφ(·), the image I ultimately produces the predicted output R.

### 2.3. Causal Data Augmentation Module

In the image frequency domain, the mid-frequency components predominantly capture domain-invariant causal features, whereas the extremely high and low frequencies contain a large proportion of domain-related non-causal features [19]. To address this, this paper introduces a CDA module based on the SCG principle [20]. This module applies Gaussian-distribution-based randomization to the frequency components of non-causal features, enhancing the diversity of source-domain data while preserving the causal features of the images. Moreover, it provides a prior foundation [21] for subsequent models to eliminate non-causal factors within the feature space.

The implementation of this module proceeds as follows. First, the discrete cosine transform (DCT) is applied to the input image to map it into the frequency domain. Next, a bandpass filter is used to identify and extract non-causal frequency components. The non-causal components are then randomly scaled according to a Gaussian distribution. Finally, the permuted non-causal spectrum is recombined with the retained causal spectrum, and the enhanced image with controlled non-causal perturbations is reconstructed via the inverse discrete cosine transform (IDCT). This procedure can be formally expressed as:

(1) $$\hat{x}\;={\;F}^{-1}(R_{N}\left(M(R\right)\cdot F(x))\;+\;(1\;-\;M(R))\cdot F(x))$$

Here, x and $\hat{x}\;$ respectively represent the input image and out images, and R represents the frequency domain. F(⋅) and $F^{-1}(\cdot )$ respectively represent the discrete cosine transform and its inverse. M(⋅)  represents the bandpass filter function, and $R_{N}(\cdot )$ denotes the Gaussian-distribution-based randomization operation. This approach effectively expands the distribution of the training data and provides essential inputs for subsequent feature debiasing within the model.

**Figure 3:**
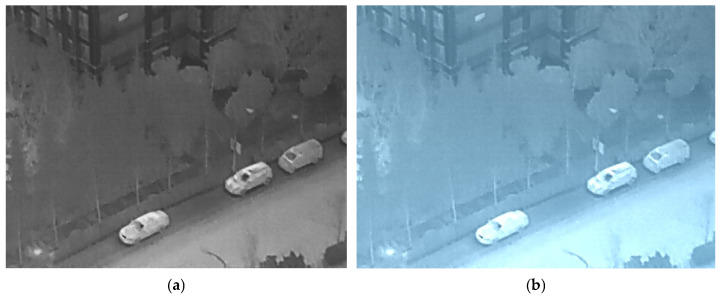
Sample images of input and output images. (**a**) The Input Image; (**b**) The Output Image.

As shown in Figure 3, the left side is the original input image, and the right side is the output image processed by the CDA module.

### 2.4. Counterfactual Reasoning Module

In infrared UAV scenes with highly similar backgrounds, excessive attention to the background can easily introduce judgment bias. Establishing an appropriate causal relationship between attention and the targets helps guide the model to distinguish causal from non-causal features in the image. This alleviates bias arising from spurious statistical associations during attention learning [22]. Existing studies [23,24,25,26,27] show that overreliance on non-causal features, such as scene information, can significantly degrade model performance. Therefore, it is necessary to design an attention adjustment mechanism that directs the model toward target-related causal features while effectively suppressing interference from non-causal factors such as the scene. This helps maintain a balanced and efficient range of feature perception.

In infrared UAV scenes, the distribution of image data is easily influenced by variations in geographical regions, imaging equipment, and environmental conditions. Annotation noise is also relatively common [28]. Inspired by contrastive attention learning [29], this paper introduces a counterfactual reasoning mechanism to enhance the model’s attention learning under such complex conditions. The core idea is to compare the effects of factual attention and counterfactual attention on the final prediction results. Factual attention refers to the attention patterns learned by the model, whereas counterfactual attention represents hypothetical patterns that do not focus on the target of interest. By analyzing the difference between these two forms of attention, the model is encouraged to improve attention quality. Maximizing this difference reduces reliance on non-causal features and leads to more robust detection performance. Unlike traditional attention mechanisms, this approach constructs counterfactual samples to intervene in the feature distribution by hypothetically removing or altering specific attention regions. This process helps identify and strengthen feature regions that are truly causally related to the prediction results. It also reduces dependence on spurious statistical associations and enhances feature discrimination in complex backgrounds.

**Figure 4:**
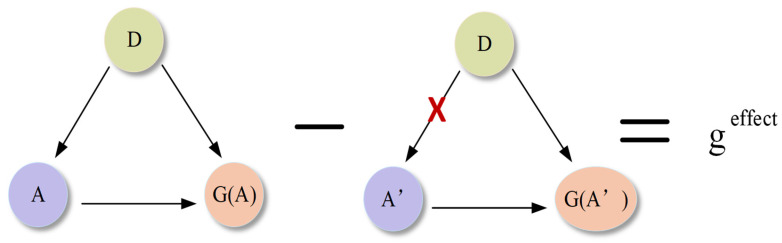
Schematic diagram of counterfactual reasoning.

As shown on the left side of Figure 4, the causal structure underlying the counterfactual reasoning mechanism involves three core variables: the input feature maps D, the regular attention feature maps A, and the actual features G(A) extracted based on A. The path D→A represents the generation of A from D, while the path (D,A) → G(A) indicates that D and A jointly determine the final extracted features G(A). In this framework, D is the causal parent of A, and G(A) is the causal child of both D and A. Ideally, A should capture the essential target-related attributes in D, such as shape, and accurately guide the extraction of G(A) for prediction. However, in practice, D often contains distracting regions. This may cause the network to converge to a suboptimal solution. To assess and improve the effectiveness of attention learning, this paper introduces causal intervention for analysis. As shown on the right side of Figure 4, the intervention do(A = A′) fixes A as the counterfactual attention feature map A′. This operation cuts off the causal path D→A′, preventing D from influencing A′. The extracted counterfactual features G(A′) can therefore be regarded as biased features. By computing the difference between G(A) and G(A′), the pure causal effect $g^{\mathrm{effect}}$ of attention learning on feature extraction can be obtained. This mechanism directly quantifies the specific contribution of attention optimization to the final features, rather than relying solely on comparisons with suboptimal outcomes.

**Figure 5:**
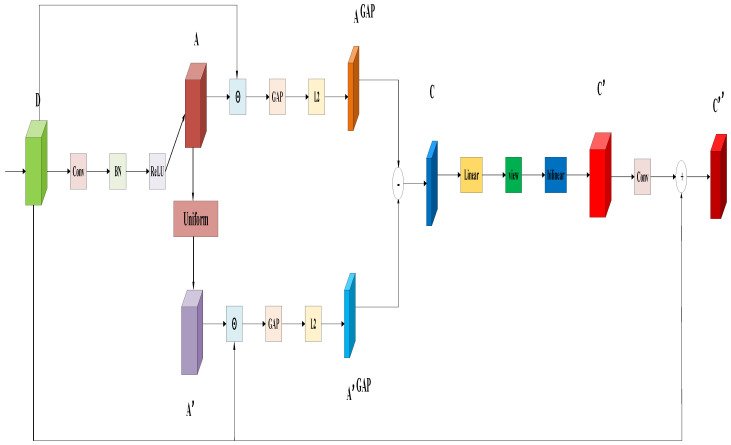
Structure diagram of the CCR module.

As shown in Figure 5, this paper proposes a CCR module based on the mechanism described above. The module is designed to establish a causal relationship between feature representations and the targets. By introducing a contrastive learning paradigm, it encourages the model to capture discriminative causal feature regions from the differences between regular attention maps and counterfactual attention maps. This leads to goal-oriented and refined attention learning. The CCR module first generates regular and counterfactual feature vectors from the corresponding attention feature maps. It then extracts causal feature vectors through contrastive learning and performs spatial reconstruction. Finally, the enhanced feature maps are output through a residual connection. In this way, the module guides the model to identify and strengthen causal features that are closely related to the targets. The detailed implementation process is described as follows:

The first step is to input the feature maps and generate the regular attention feature maps through the convolutional layer, batch normalization layer and activation function layer. The specific process is as follows:

(2) $$A\;=\;\mathrm{ReLU}(\mathrm{BN}\left(\mathrm{Conv}\left(D\right)\right))$$

Here, D represents the input feature maps, Conv represents the convolutional layer, BN represents the batch normalization layer, ReLU represents the activation function layer, and A represents the regular attention feature maps.

In the second step, during the training stage, a counterfactual attention feature map is generated by applying random perturbations to the regular attention feature map. The perturbation factors follow a uniform distribution over the interval [0, 2]. During the testing stage, the random perturbations are removed and the disturbance factor is fixed at 1. In this case, the regular attention feature map is directly used for prediction. The specific process is as follows:

(3) $$\begin{array}{cccc} & A\prime \;=\;\left\{\begin{array}{cc} \mathrm{Uniform}(0,2)(A), & the\;training\;stage\\ 1(A), & the\;testing\;stage \end{array}\right. & & \end{array}$$

Here, A denotes the regular attention feature map. Uniform(0,2) indicates a uniform distribution over the interval [0, 2]. The value 1 means that no perturbation is applied. A′ represents the counterfactual attention feature map generated after perturbation.

In the third step, the regular attention feature map and the counterfactual attention feature map are applied separately to the input feature map. Element-wise weighting is first performed, followed by global average pooling and L2 normalization. This process yields the weighted, pooled, and normalized regular feature vector and counterfactual feature vector, respectively. The specific procedure is as follows:

(4) $$A^{\mathrm{GAP}}\;=\;L2(\mathrm{GAP}\left(A⊙D\right)),{A^{\prime }}^{\mathrm{GAP}}\;=\;L2(\mathrm{GAP}(A\prime ⊙D))$$

Here, D represents the input feature maps, ⊙ indicating element-by-element weighting, A represents the regular attention feature maps, A’ represents the counterfactual attention feature maps, GAP represents global average pooling in the spatial dimension (height × width), L2 represents L2 normalization, $A^{\mathrm{GAP}}$ and $\;A^{\prime \mathrm{GAP}}$ respectively represent the weighted, pooled, and normalized regular and counterfactual feature vectors.

In the fourth step, causal feature vectors are extracted through contrastive learning within the weighted, pooled, and normalized regular and counterfactual feature vector spaces. Specifically, contrastive learning removes spurious correlations by computing the differences between the regular feature vectors and the counterfactual feature vectors, while preserving the truly discriminative causal feature vectors. The detailed process is as follows:

(5) $${C\;=\;A}^{\mathrm{GAP}}\;-{\;A}^{\prime \mathrm{GAP}}$$

Here, $A^{\mathrm{GAP}}$ and $A^{\prime \mathrm{GAP}}\;$ respectively represent the weighted, pooled, and normalized regular and counterfactual feature vectors, and C represents the causal feature vectors.

In the fifth step, the causal feature vectors are reconstructed into causal feature maps through a linear layer, followed by reshaping and bilinear interpolation. This process restores the spatial structural information of the features. The specific procedure is as follows:

(6) $$C^{\prime }=\mathrm{bilinear}(\mathrm{view}(\mathrm{Linear}(C)))$$

Here, C denotes the causal feature vector. Linear represents the linear layer. View indicates the flattening operation, and bilinear denotes bilinear interpolation. C′ represents the reconstructed causal feature map.

In the sixth step, the output channels of the causal feature maps are adjusted using a convolutional layer. A residual connection with the input feature maps is then applied to produce the enhanced feature maps. The specific procedure is as follows:

(7) $$C\prime \prime \;=\;D\;+\;\mathrm{Conv}(C^{\prime })$$

Here, C′ represents the causal feature maps, Conv represents the convolutional layer, + represents the residual connection, D represents the input feature maps, and C″ represents the enhanced feature maps.

### 2.5. Causal Attention Mechanism Module

The attention mechanism [30], a core component of the Transformer architecture, models long-range dependencies globally by computing association weights between any two positions or feature vectors in the input sequence. In visual tasks, it has been successfully applied to sequences of image patches. Each patch, after linear projection, serves as a sequence element, allowing the model to dynamically aggregate contextual information relevant to a specific query region [3]. The standard computation process can be formally expressed as:

(8) $$\mathrm{Attention}(Q,K,V)\;=\;\mathrm{Softmax}\left(\frac{QK^{T}}{\sqrt{d_{k}}}\right)V$$

Here, the left Attention(Q,K,V) represents the attention function, which accepts three inputs: the query matrix Q, the key matrix K, and the value matrix V. The right query matrix Q, key matrix K, and value matrix V are respectively obtained through linear projection $W^{Q},W^{K},W^{V}$ of the input feature sequence. ${\mathrm{QK}}^{T}$ represents the calculation of the similarity between the query matrix and the key matrix to obtain the attention score matrix. $d_{k}$ represents the dimension of the key vector and $\sqrt{d_{k}}$ represents the scaling factor, which is used to prevent the dot product from being too large and causing the gradient to disappear. Here, Softmax indicates that each row of the attention score matrix is normalized to produce the attention weight matrix. When Q, K, and V are all derived from the same input feature sequence, the mechanism is called self-attention. When they come from different input feature sequences, it is referred to as cross-attention. This design enables the model to perform powerful feature interactions and information filtering, achieving state-of-the-art performance across numerous visual benchmarks.

The performance gains of the attention mechanism largely rely on the strong assumption that the training and testing data follow an independent and identically distributed (IID) pattern [31]. Under this assumption, the attention weights learned by the model essentially reflect the statistical correlations between input features. In complex infrared UAV scenarios, if a particular background frequently co-occurs with targets in the training data, the mechanism tends to assign it higher weight, reinforcing the correlation between the background and the targets. Even though this correlation-based learning can be effective and efficient for prediction, it has limitations.

In real-world deployment, out-of-distribution (OOD) situations are common. In such cases, the statistical correlations learned under the IID assumption become fragile. Background features that originally co-occurred with targets may act as confounding factors, forming pseudo-correlations that interfere with predictions in new environments. For example, a model trained to associate birds with the ground may be misled when it encounters a rare scenario, such as bears standing on the ground, resulting in incorrect predictions. This highlights a fundamental limitation of traditional attention mechanisms: they cannot identify the causal direction underlying feature associations. Consequently, they are unable to distinguish whether relationships between features arise from stable causal mechanisms or merely from statistical contingencies in the training data.

The ideal attention mechanism should identify and emphasize causal features that are truly essential for prediction while suppressing non-causal features that are merely statistically correlated but have no causal effect. Therefore, it is necessary to shift the attention mechanism from focusing on correlation to focusing on causality. To achieve this, this paper proposes a CAM module. Its core objective is to allocate attention weights based solely on the true causal effects between features and targets. By distinguishing and weighting causal and non-causal features, the module guides the model to concentrate on features that are genuinely relevant to the targets. The specific implementation process is as follows:

In the first step, the input feature sequence is simultaneously projected into joint query, key, and value matrices using a shared linear projection layer. These matrices are then reshaped and split into multiple attention heads, producing the final multi-head query, key, and value matrices. The specific process is as follows:

(9) $$\begin{array}{cccc} & \left[Q,K,V\right]\;=\;\mathrm{ReshapeToHeads}\left(XW^{\mathrm{QKV}}\right) & & \end{array}$$

Here, X represents the input feature sequence, $W^{\mathrm{QKV}}$ represents the linear projection layer, ReshapeToHeads represents reshaping and multi-head operations, and Q, K, and V respectively represent the query, key, and value matrices.

The second step is to calculate two complementary attention weight matrices in parallel. The specific process is as follows:

(10) $$\;\;\;\begin{array}{cccc} & \;\;\;\;\;A_{c}\;=\;\mathrm{Softmax}\left(\frac{QK^{⊤}}{\sqrt{d_{k}}}\right) & & \end{array}$$

(11) $$\begin{array}{cccc} & \;\;\;\;\;A_{s}=\mathrm{Softmax}\left(-\frac{QK^{⊤}}{\sqrt{d_{k}}}\right) & & \end{array}$$

Here, Q and K represent the query and key matrices, respectively. ${\mathrm{QK}}^{T}$ computes the similarity between the query and key matrices to obtain the attention score matrix. $d_{k}$ denotes the dimension of the key vector, and $\sqrt{d_{k}\;}\;$ serves as a scaling factor to prevent the dot product from becoming too large and causing gradient issues. Softmax normalizes each row of the attention score matrix to produce the attention weight matrix. $A_{c}$ represents the causal attention weight matrix, with high-weight regions highlighting features that are potentially causally related to the targets. $A_{s}$ represents the non-causal attention weight matrix. By taking the negative of $A_{s}$, the high-weight regions are forced to focus on background patterns ignored by $A_{c}$, explicitly modeling spurious associations.

In the third step, the two computed attention weight matrices are applied separately to the value matrix. This yields the initially decoupled causal and non-causal features. The specific process is as follows:

(12) $$\begin{array}{cccc} & X_{c}\;=\;A_{c}V & & \end{array}$$

(13) $$\begin{array}{cccc} & X_{s}={\;A}_{s}V & & \end{array}$$

Here, $X_{c}$ represents the initially decoupled causal features, and $X_{s}$ represents the initially decoupled non-causal features. Linear denotes the linear layer, and Dropout represents the regularization layer. $X_{c}^{\prime }$ and $X_{s}^{\prime }$ are the resulting transformed causal and non-causal features, respectively.

In the fourth step, the initially decoupled causal and non-causal features are each passed through a shared linear layer followed by a regularization layer. This performs feature transformation and dimension adjustment, yielding the transformed causal and non-causal features. The specific process is as follows:

(14) $$\begin{array}{cccc} & {\;X}_{c}^{\prime }\;=\;\mathrm{Dropout}(\mathrm{Linear}(X_{c})) & & \end{array}$$

(15) $${\;X}_{s\;}^{\prime }=\mathrm{Dropout}(\mathrm{Linear}(X_{s}))$$

Here, $X_{c}$ represent the initially decoupled causal features, $X_{s}\;$ represent the initially decoupled non-causal features, Linear represents the linear layer, Dropout represents the regularization layer, $X_{c}^{\prime }\;$ represent the transformed causal features, and $X_{s}^{\prime }\;$ represent the transformed non-causal features.

In the fifth step, a residual connection is first applied between the original input features and the transformed causal features. The resulting features are then processed through a normalization layer and a multi-layer perceptron to obtain the deeply decoupled causal features. The specific process is as follows:

(16) $$\begin{array}{cccc} & \;C\;=\;\mathrm{MLP}(\mathrm{LN}({X\;+\;X}_{c}^{\prime })) & & \end{array}$$

Here, X represents the original input features, $X_{c}^{\prime }\;$ represents the transformed causal features, + represents the residual connection, LN represents the normalization layer, MLP represents the multi-layer perceptron, and C represents the deeply decoupled causal features.

In the sixth step, the original input features, the transformed causal features, and the deeply decoupled causal features are combined through a residual concatenation. This produces the final essential features representing the causal associations to be extracted. The specific process is as follows:

(17) $$\begin{array}{cccc} & \;C\prime \;=\;{X\;+\;X}_{c}^{\prime }\;+\;C & & \end{array}$$

Here, X represents the original input features, $X_{c}^{\prime }\;$ represents the transformed causal features, C represents the deeply decoupled causal features, + represents the residual connection, and C′ represents the essential features of causal association.

**Figure 6:**
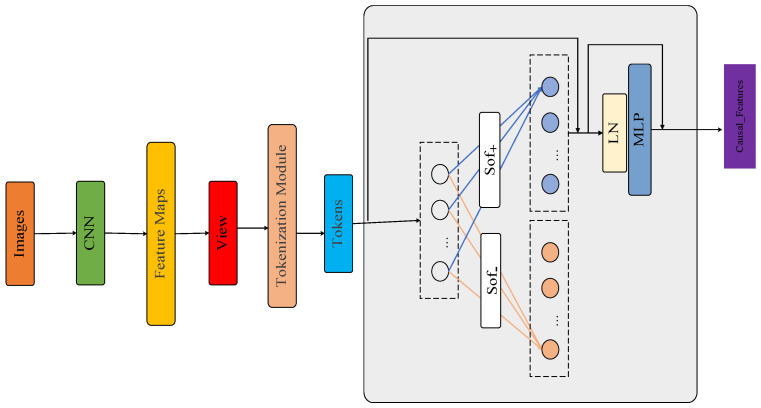
Structure diagram of the CAM module.

As shown in Figure 6, the processing flow of the CAM module is as follows. First, multi-scale feature maps are extracted from the input images using a convolutional neural network. These feature maps are then tokenized into a sequence of feature tokens through spatial flattening. After adding positional encoding to this sequence, it is fed into the core processing layer composed of stacked causal attention blocks. Within each block, features interact and are decoupled through key operations, including the causal attention mechanism with complementary Softmax functions. The resulting features then undergo nonlinear transformation and fusion via a normalization layer and a multi-layer perceptron. Finally, the module outputs the causal feature representation, Causal_Features, completing the end-to-end mapping from the original images to a robust feature representation. Each component is sequentially connected, forming a hierarchical feature processing pathway.

## 3. Experimental Setup and Implementation Details

### 3.1. Dataset

**Figure 7:**
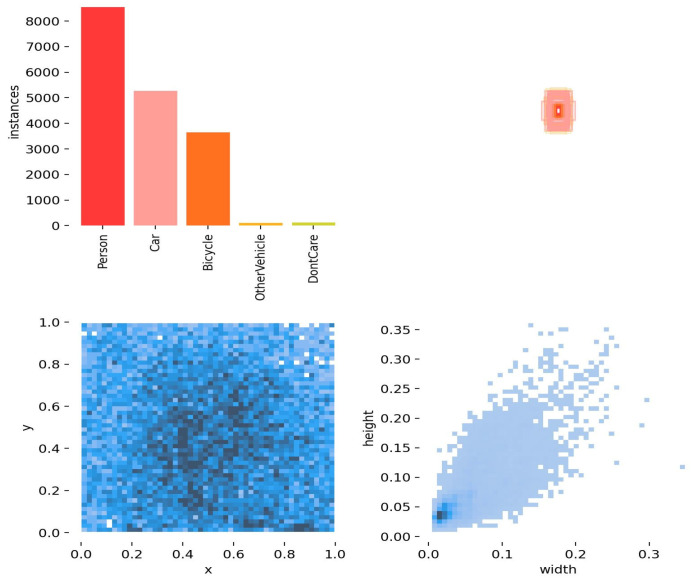
Category Distribution Map of HIT-UAV dataset.

This experiment uses the HIT-UAV high-altitude infrared thermal imaging UAV dataset, which was publicly released in March 2023. The dataset is derived from 43,470 frames of UAV video and includes metadata such as flight altitude, camera perspective, and lighting conditions, providing a valuable resource for low-altitude infrared target detection on UAV platforms. In this study, 2029 images were used for training and 290 for validation. To ensure compatibility with different detection tasks and algorithm frameworks, the dataset was converted to the MS COCO annotation format with standard bounding box annotations. This format supports accurate detection in scenarios involving target overlap and occlusion from multiple perspectives at high altitudes. Both the training and validation sets underwent annotation alignment and format standardization, achieving a 100% data integrity retention rate. This provides a reliable foundation for research on infrared UAV small target detection. The detailed category distribution statistics are shown in Figure 7.

As shown in Figure 7, the upper bar chart presents the number of instances in each category within the dataset. The person category has significantly more instances than other categories, indicating the presence of category imbalance. The heat map on the lower left illustrates the spatial distribution of targets along the x and y coordinates, showing that targets are primarily concentrated in the central region of the images. The scatter plot on the lower right depicts the relationship between target width and height, revealing that small targets tend to cluster within a certain size range. Overall, this dataset exhibits three key characteristics: category imbalance, concentrated spatial distribution, and aggregation of small target sizes. These properties provide an important data foundation for the design and optimization of small target detection algorithms.

**Figure 8:**
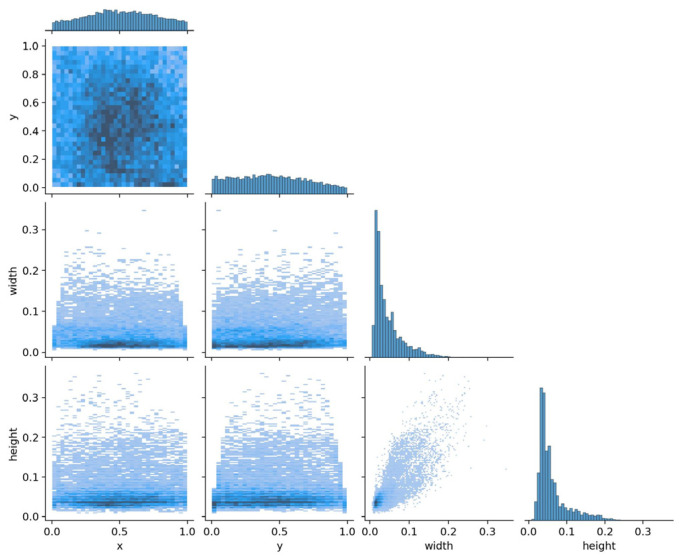
Joint visualization Diagram of the target dimension distribution of the HIT-UAV dataset.

As shown in the joint visualization graph in Figure 8, the distribution characteristics of targets in the HIT-UAV dataset are presented across four dimensions: center coordinates (x,y), width, and height. The histograms along the diagonal display the marginal distributions of each variable. The x and y coordinates are approximately evenly distributed, indicating broad coverage of the target space. In contrast, the width and height distributions are right-skewed, reflecting the predominance of small-sized targets. The scatter plots in the off-diagonal positions reveal relationships between variables. The x and y coordinates are concentrated near the center of the images. Width and height show no obvious association with x or y, but they are positively correlated with each other, which aligns with the realistic width-to-height ratios of the targets.

### 3.2. Evaluation Indicators

In this experiment, mAP@50 and mAP@50:95 are used as evaluation metrics for model performance. mAP@50 is a fundamental metric in object detection, reflecting the mean Average Precision (mAP) when the Intersection over Union (IoU) threshold is 0.5. Its value provides an intuitive measure of the model’s performance under standard matching criteria. mAP@50:95 is the arithmetic mean of mAP values computed across IoU thresholds ranging from 0.5 to 0.95, in steps of 0.05. This metric comprehensively reflects the model’s performance under varying localization accuracy requirements and serves as an important indicator of the detector’s robustness.

The calculation formula of mAP is:

(18) $$\;P\;=\;\frac{\mathrm{TP}}{\mathrm{TP}+\mathrm{FP}}$$

(19) $$\;\;R=\frac{\mathrm{TP}}{\mathrm{TP}+\mathrm{FN}}\;$$

(20) $$P_{\mathrm{AP}}=\int_{0}^{1}P(R)\mathrm{dR}$$

(21) $$P_{\mathrm{mAP}}=\frac{1}{C}\sum_{i=1}^{c}P_{{\mathrm{AP}}_{i}}$$

In the formula: TP represents the number of samples that the model correctly predicts as positive classes; FP represents the number of samples that the model wrongly predicted as positive classes; FN represents the number of positive class samples that the model wrongly predicted as negative classes; P indicates the precision rate; R indicates the recall rate; $P_{\mathrm{AP}}$ represents the average precision value calculated for a single category in the dataset under a certain specific IoU threshold; C represents the total number of categories in the dataset; $P_{\mathrm{mAP}}$ represents the arithmetic mean of the average precision values of all categories in the dataset at a certain specific IoU threshold.

### 3.3. Experimental Environment and Parameter Settings

This experiment is conducted using the PyTorch 2.1 deep learning framework within a Python 3.11 environment, accelerated by CUDA 12.1 and cuDNN 8.8.1. All code runs on a Windows 11 server equipped with an Intel Core i9-13900K processor and an NVIDIA GeForce RTX 4090 GPU. The specific training configuration is as follows: training is performed for 300 epochs with a batch size of 4, and input images are uniformly resized to 640 × 640 pixels. The AdamW optimizer is used, with an initial learning rate of 0.0001, a momentum coefficient of 0.9, and a weight decay of 0.0001. The overall loss function consists of four components: bounding box regression loss, classification loss, target confidence loss, and distribution focus loss, with weight coefficients of 7.5, 0.5, 1.0, and 1.5, respectively. This consistent training configuration provides a reliable basis for fair performance comparisons across different models.

## 4. Experimental Results and Analysis

### 4.1. Analysis of the Results of the Confusion Matrix Graph

**Figure 9:**
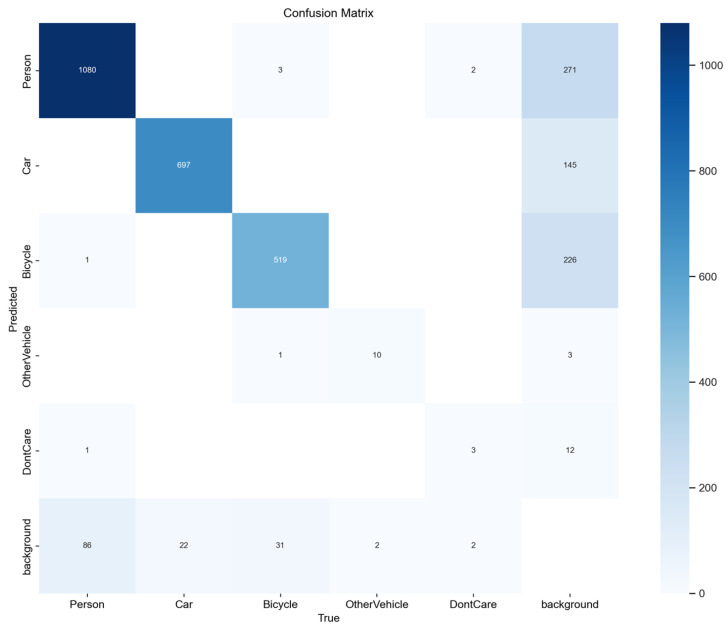
Confusion matrix diagram.

As shown in the confusion matrix in Figure 9, the model’s detection performance during training is clearly illustrated. From the matrix, the model demonstrates strong recognition for common categories such as Person, Car, and Bicycle, indicated by higher values along the diagonal. However, its detection performance for low-frequency categories, such as OtherVehicle and DontCare, is relatively weak. The matrix also reveals two notable issues. First, there are false detections between the background and foreground categories (e.g., nonzero values in the background row corresponding to other categories), indicating that confusion between background and foreground targets is significant. Second, mutual misclassifications among certain categories (e.g., non-diagonal values) reflect insufficient differentiation between categories.

In summary, the model’s performance is primarily constrained by three factors: uneven sample distribution across categories, insufficient differentiation between foreground and background, and unclear feature representation among similar categories. Future optimization can focus on three directions: balancing category samples through data augmentation and related techniques, enhancing the feature extraction module to better separate foreground from background, and incorporating a more refined category feature learning mechanism to improve discrimination among easily confused categories. These improvements can collectively enhance the model’s detection accuracy and stability.

### 4.2. F1–Confidence Analysis of the Curve Graph Results

**Figure 10:**
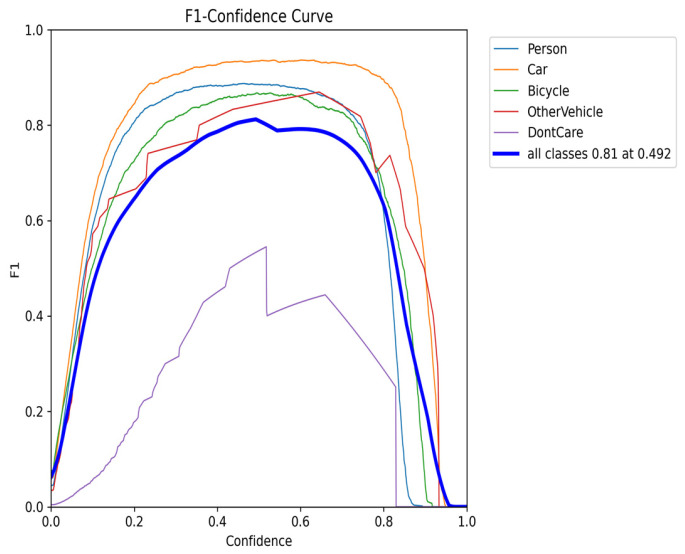
F1-Confidence Curve Graph.

As shown in Figure 10, the F1–Confidence curve illustrates how the F1 scores of different categories vary across confidence thresholds during training. The model maintains high F1 scores for common categories such as Person, Car, and Bicycle over a wide confidence range, indicating a good balance between precision and recall for these categories. In contrast, the curves for categories like DontCare remain relatively low, reflecting weaker detection performance. From an overall perspective, the peak average F1 score across all categories is 0.81, occurring at a confidence threshold of 0.492. However, this value leaves room for improvement due to lower performance in certain categories. Moreover, when the confidence threshold exceeds 0.8, the F1 score drops sharply, indicating that the model’s high-confidence predictions are not yet fully reliable.

In summary, the model’s performance is primarily limited by three factors: large performance disparities across categories, weak recognition for certain categories (such as DontCare), and insufficient stability in high-confidence predictions. Future optimization can focus on three directions: augmenting data for underrepresented categories to reduce distribution imbalance, applying confidence calibration techniques to improve the reliability of high-confidence predictions, and enhancing the model’s balanced learning across categories through loss function weighting. These strategies aim to improve the overall robustness of the model across different confidence levels.

### 4.3. Analysis of the Results of P-Confidencee, R-Confidencee, and PR Curve Graphs

**Figure 11:**
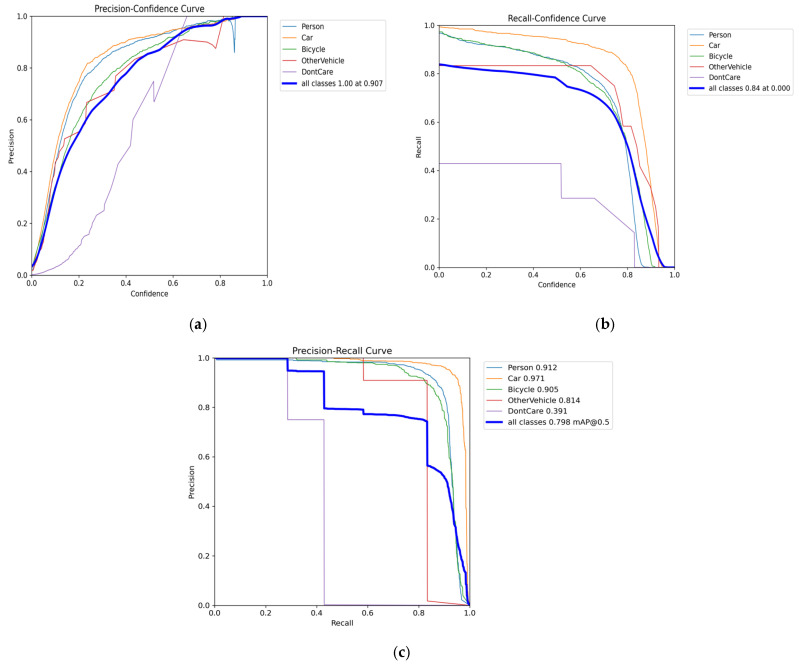
Curves Graph of P, R and PR. (**a**) P-Confidencee Curves Graph; (**b**) R-Confidencee Curves Graph; (**c**) PR Curves Graph.

As shown in Figure 11, the performance of the target detection model in key categories (Person, Car, Bicycle) and as a whole during the training stage was evaluated from multiple dimensions through three types of performance curves: precision-confidence, recall-confidence, and precision-recall:

(1) Precision-Confidence Curve: As shown in Figure 11a, the precision of most categories, such as Person, Car, and Bicycle, increases steadily with rising confidence levels, which aligns with reasonable expectations for model performance. However, the curve of the DontCare category fluctuates significantly and is generally low, indicating that the model’s prediction reliability in this category is weak and the performance balance among categories is insufficient.
(2) Recall-Confidence Curve: As shown in Figure 11b, the overall recall decreases as the confidence threshold increases, consistent with theoretical expectations. However, the recall for DontCare remains at a relatively low level throughout, while the recall for major categories such as Person and Car drops sharply when the confidence level exceeds 0.8. This suggests a significant risk of missed detections for mainstream categories at high confidence levels.
(3) Precision-Recall Curve: As shown in Figure 11c, categories such as Person, Car, and Bicycle have relatively large areas under the curve, indicating a good balance between precision and recall. In contrast, the DontCare curve decays rapidly and has a very small area, and the OtherVehicle category also shows weak performance. This highlights the model’s difficulty in maintaining both recognition accuracy and recall coverage for certain categories.

In summary, the model exhibits three main performance issues: large performance disparities among categories, a high risk of missed detections for mainstream categories at high confidence levels, and difficulty balancing accuracy and recall for weak categories. Future optimization can focus on three strategies: alleviating category imbalance through data augmentation, applying confidence calibration to reduce missed detections at high thresholds, and designing weighted loss functions for underrepresented categories to improve overall balance and reliability.

### 4.4. Analysis of the Results of the Loss Function and Evaluation Index Curve Graph

**Figure 12:**
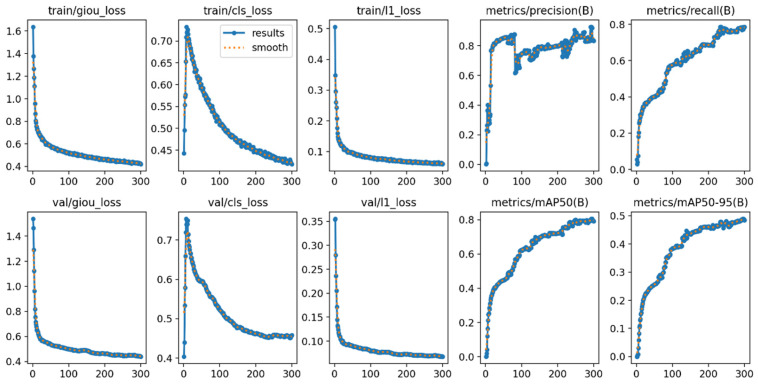
Curve graph of the loss function and evaluation indicators.

As shown in Figure 12, the loss and evaluation metric curves during the model training process demonstrate good convergence performance. The various losses for the training and validation sets including giou_loss, cls_loss, and l1_loss decreased rapidly at the beginning of the iterations and gradually stabilized, indicating that the model parameters were effectively optimized. Meanwhile, the precision, recall, and core evaluation metrics such as mAP@50 and mAP@50:95 steadily increased with each training round and eventually reached saturation, demonstrating continuous improvement and stable convergence in detection accuracy, recall ability, and overall localization performance. Furthermore, the loss curves of the training and validation sets show consistent trends without significant deviations, indicating that no notable overfitting occurred during training. This further validates the effectiveness of the training process and the reliability of the model’s performance.

### 4.5. Comparative Experimental Results Analysis

As illustrated in Table 1, this experiment systematically evaluated various mainstream target detection models, including DETR and Deformable-DETR, alongside the CSFPR-RTDETR series models introduced in this article, using the HIT-UAV public dataset. The results highlight the trade-offs among model size, efficiency, and accuracy. Regarding model parameters and FLOPs, CSFPR-RTDETR (14.1M, 63.8G) and CSFPR-RTDETR-CR (15.2M, 64.6G) have significantly lower parameter counts and computational demands compared to DETR (41.0M, 86.0G), Deformable-DETR (40.0M, 173.0G), and other models, while remaining slightly higher than the lightweight D-FINE and DEIM (both 4.0M, 7.0G). In terms of detection accuracy, the proposed CSFPR-RTDETR-CR achieves outstanding performance, with mAP@50 of 81.2% and mAP@50:95 of 51.5%, surpassing classical models such as DETR and Deformable-DETR. Additionally, both CSFPR-RTDETR (9.6 ms) and CSFPR-RTDETR-CR (9.6 ms) maintain excellent real-time performance, slightly slower than RT-DETR (7.6 ms) but still highly efficient.

### 4.6. Analysis of Ablation Experiment Results

In order to analyze the contribution of each module in the CSFPR-RTDETR-CR model, this paper designed a systematic ablation experiment. The configuration and results are shown in Table 2.

(1) Baseline model (CSFPR-RTDETR): All metrics are used as the reference baseline.
(2) Introducing the CDA module (CSFPR-RTDETR + CDA): The parameters and FLOPs remain unchanged. mAP@50 decreases by 0.7%, mAP@50:95 decreases by 1.9%, and inference time is slightly reduced by 0.1 ms. This indicates that while the CDA module enhances sample diversity, it also introduces additional non-causal features, which slightly reduces detection accuracy but has minimal impact on model complexity and speed.
(3) Introducing the CCR module (CSFPR-RTDETR + CCR): The parameters increase slightly by 0.6M and FLOPs by 0.1G. mAP@50 improves by 2.9% and mAP@50:95 by 0.9%. Meanwhile, inference time is reduced by 0.4 ms, demonstrating that the CCR module effectively enhances the causal features of the detection targets and contributes to improved performance.
(4) Introducing the CAM module (CSFPR-RTDETR + CAM): The parameters increase slightly by 0.5M, and FLOPs increase by 0.7G. mAP@50 improves by 1.9% and mAP@50:95 by 1.5%, while inference time is slightly reduced by 0.1 ms. This demonstrates the effectiveness of the CAM module in guiding the model to focus on the causal features of detection targets.
(5) CSFPR-RTDETR-CR model (CSFPR-RTDETR + CDA + CCR + CAM): After integrating the CDA, CCR, and CAM modules, the parameters and FLOPs increase by 1.1M and 0.8G, respectively, while inference time remains unchanged. The model achieves the highest values in core metrics, mAP@50 and mAP@50:95, confirming the collaborative advantage of combining multiple modules. This indicates that the modules complement each other and jointly produce optimal performance improvement.

### 4.7. Heat Maps Visual Analysis

**Figure 13:**
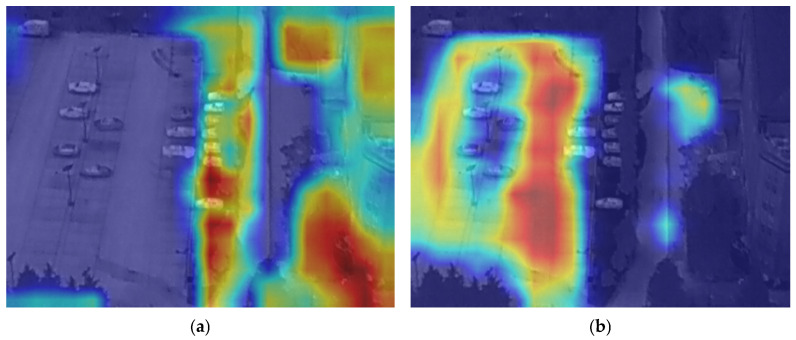
Heat Maps of the CSFPR-RTDETR and CSFPR-RTDETR + CCR models. (**a**) CSFPR-RTDETR; (**b**) CSFPR-RTDETR + CCR.

As shown in Figure 13, the two heat maps illustrate the attention effects of different models on the target area. Figure 13a presents the thermal distribution of the CSFPR-RTDETR model. Its attention is concentrated on the right area, but the range is relatively narrow, and coverage in some regions (such as the left) is uneven. Figure 13b shows the CSFPR-RTDETR + CCR model after integrating the CCR module. The thermal distribution is broader and more layered, enhancing attention intensity in key areas (with red regions more concentrated) and providing more comprehensive coverage of surrounding relevant areas. This indicates that the CCR module improves both the completeness and accuracy of the model’s attention to the target region.

**Figure 14:**
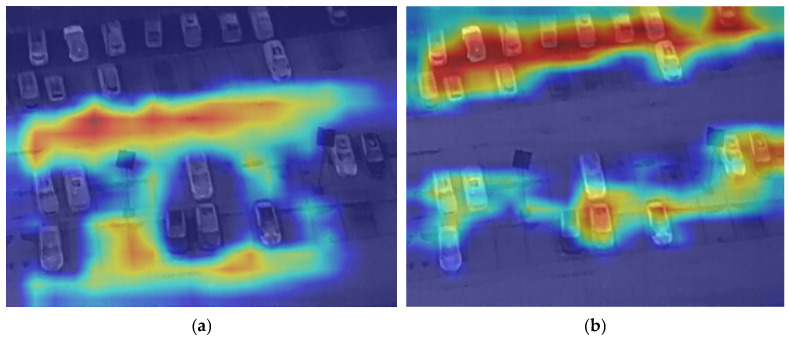
Heat Maps of the CSFPR-RTDETR and CSFPR-RTDETR + CAM models. (**a**) CSFPR-RTDETR; (**b**) CSFPR-RTDETR + CAM.

As shown in Figure 14, the two heat maps illustrate the attention effects of different models on the target area. Figure 14a shows the thermal distribution of the CSFPR-RTDETR model, where the attention is relatively scattered and lacks concentration. The red high-attention regions cover a wide area but show weak focus on individual vehicles. Figure 14b shows the CSFPR-RTDETR + CAM model after adding the CAM module. Its thermal distribution is more concentrated on each vehicle, with the red high-attention areas closely aligned with the vehicle positions and reduced redundant attention in irrelevant regions. This indicates that the CAM module improves the model’s positioning accuracy and enhances targeted attention on the objects.

### 4.8. Frequency-Domain Feature Maps Visual Analysis

**Figure 15:**
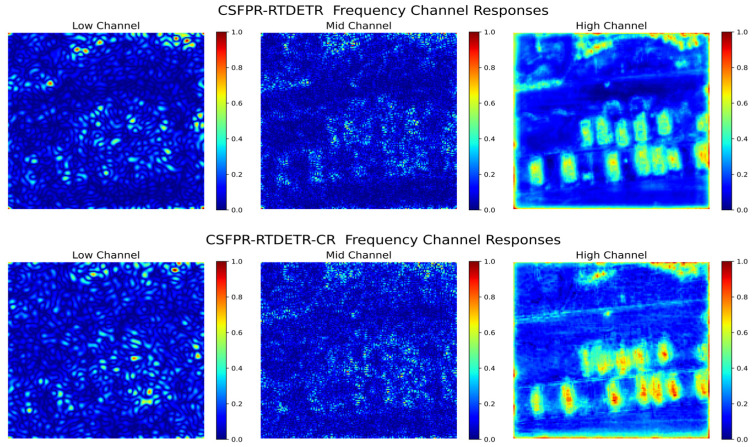
Frequency-Domain feature maps of the CSFPR-RTDETR and CSFPR-RTDETR-CR models.

As shown in Figure 15, the figure compares the feature responses of the CSFPR-RTDETR baseline model and the proposed model across low, medium, and high frequency channels. Although the high-frequency channels of the CSFPR-RTDETR model show some target-related responses, the intensity is weak and the boundaries are blurred, while the responses in the low- and medium-frequency channels are more scattered. In contrast, the high-frequency response of the CSFPR-RTDETR-CR model is more focused on the target area, with brighter and more concentrated colors, and the responses in the low- and medium-frequency channels are more regular. This indicates that the proposed model enhances the ability to capture target features across all frequency channels, resulting in more accurate and targeted representation of frequency-domain features.

### 4.9. Detection Results Visual Analysis

**Figure 16:**
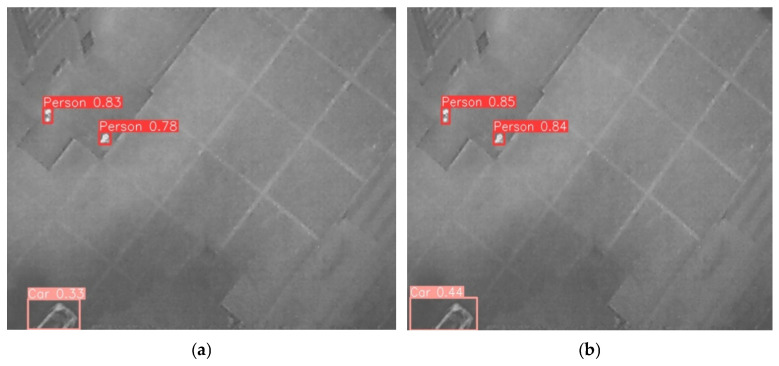
Visualization of the detection results of the CSFPR-RTDETR and CSFPR-RTDETR-CR models. (**a**) CSFPR-RTDETR; (**b**) CSFPR-RTDETR-CR.

As illustrated in Figure 16, a comparison of detection results between the baseline CSFPR-RTDETR model and the proposed CSFPR-RTDETR-CR model is conducted in infrared UAV scenarios. Notable differences are observed in both target recognition confidence and completeness. The CSFPR-RTDETR model, shown in Figure 16a, detected two Person targets with confidence levels of 0.83 and 0.78, respectively, and one Car target with a confidence level of 0.33. In contrast, the CSFPR-RTDETR-CR model, shown in Figure 16b, not only maintains the same detection coverage but also increases the confidence of the Car target to 0.44 and raises the confidence levels of the two Person targets to 0.85 and 0.84, respectively. These results indicate that the CSFPR-RTDETR-CR model has superior feature extraction and target perception capabilities, effectively improving the completeness and reliability of detection outcomes.

## 5. Conclusions

To address the issue of insufficient model generalization resulting from the learning of spurious statistical associations in the small target detection task of infrared UAV, this paper presents an enhanced framework termed A Triple Path Causal Intervention, grounded in causal reasoning theory. This framework utilizes the advanced CSFPR-RTDETR detector as a baseline and explicitly decouples causal and non-causal features within the feature space, guided by a structural causal model. It systematically mitigates feature bias through three distinct paths. First, a CDA module is designed to apply frequency perturbations based on a Gaussian distribution to non-causal features, thereby bolstering the model’s robustness against background disturbances. Second, a CCR module is embedded within the backbone network to improve the recognition of causal features by generating counterfactual samples. Third, a CAM module is introduced into the encoder, which explicitly distinguishes and weights causal and non-causal features, encouraging the model to concentrate on the causal information pertinent to the target. Experiments conducted on the HIT-UAV public dataset demonstrate that this framework yields improvements of 5.6% and 1.8% in the core metrics of mAP@50 and mAP@50:95, respectively. Visual analyses further corroborate its effectiveness in enhancing feature discrimination.

This study acknowledges that the current framework has limitations in computational efficiency and in verifying generalization. Future research will focus on two main directions. First, lightweight model design: by applying techniques such as structural pruning and knowledge distillation, we aim to reduce computational overhead while maintaining performance, thereby improving deployment on embedded devices. Second, cross-domain generalization: systematic testing on a wider range of infrared UAV datasets will be conducted to further evaluate the framework’s robustness and adaptability.

Although further optimization is possible, the framework presented in this paper provides an effective solution that leverages causal reasoning to enhance detection performance in infrared UAV small target tasks. Moreover, it establishes a methodological foundation for deeper application of causal approaches in low-level visual tasks.

## Figures and Tables

**Table 1:** Comparative experimental results of different target detection models. Parameters, FLOPs, and Inference-time are reported in Million (M), Giga (G), and milliseconds (ms), respectively.

Method	Parameters (M)	FLOPs (G)	mAP@50 (%)	mAP@50:95 (%)	Inference-Time (ms)
DETR [32]	41.0	86.0	61.2	34.5	-
Deformable-DETR [33]	40.0	173.0	72.1	42.8	-
DINO [34]	47.0	279.0	76.7	49.6	-
Co-DETR [35]	-	-	79.1	50.0	-
RT-DETR [36]	41.9	125.6	73.7	47.6	7.6
D-FINE [37]	4.0	7.0	79.5	49.6	-
DEIM [38]	4.0	7.0	76.9	48.2	-
CSFPR-RTDETR [39]	14.1	63.8	75.6	49.7	9.6
RF-DETR [40]	30.5	31.9	62.6	37.4	-
CSFPR-RTDETR-CR	15.2	64.6	81.2	51.5	9.6

**Table 2:** The ablation experiment results of the CSFPR-RTDETR-CR model. Parameters, FLOPs, and Inference-time are reported in Million (M), Giga (G), and milliseconds (ms), respectively.

Method	Parameters 50.	FLOPs (G)	mAP@50 (%)	mAP@50:95 (%)	Inference-Time (ms)
CSFPR-RTDETR	14.1	63.8	75.6	49.7	9.6
CSFPR-RTDETR + CDA	14.1	63.8	74.9	47.8	9.5
CSFPR-RTDETR + CCR	14.7	63.9	78.5	50.6	9.2
CSFPR-RTDETR + CAM	14.6	64.5	77.5	51.2	9.5
CSFPR-RTDETR-CR	15.2	64.6	81.2	51.5	9.6

## Data Availability

No new data were created or analyzed in this study. Data sharing is not applicable to this article.
